# Effectiveness of vestibular incision subperiosteal tunnel access (VISTA) technique with or without A-PRF in treatment of multiple adjacent gingival recession defects (MAGRD): A 12 months CBCT study

**DOI:** 10.1371/journal.pone.0338823

**Published:** 2025-12-23

**Authors:** Prabhnoor Tuli, Abhay P. Kolte, Rajashri A. Kolte, Vrushali N. Lathiya, Vinisha A. Bajaj, Shahabe Saquib Abullais, Manea M. Alahmari

**Affiliations:** 1 Department of Periodontics and Implant Dentistry, Ranjeet Deshmukh Dental College & Research Centre, Nagpur, Maharashtra, India; 2 Department of Periodontics and Community Dental Science, King Khalid University, College of Dentistry, Abha, Saudi Arabia; University of Florida Jacksonville, UNITED STATES OF AMERICA

## Abstract

**Objectives:**

In order to treat MAGRD in the maxillary anterior region, the VISTA approach was evaluated and compared with and without A-PRF.

**Materials and methods:**

A split mouth RCT was designed with 216 MAGRD that were assigned to VISTA alone and VISTA with A-PRF. The complete root coverage (CRC) and gingival thickness (GT) were measured using CBCT at baseline and 12 months post-operatively, while the clinical parameters of probing depth (PD), clinical attachment level (CAL), width of keratinized gingival (WKG), gingival recession depth (GRD), and gingival recession width (GRW) were recorded at baseline, 6 months, and 12 months.

**Results:**

From baseline to 12 months, there was a significant decrease in the mean values of GRD and GRW with an increase in WKG. CBCT scans showed a significant increase in GT mean values. According to these results, the Test group’s CRC was higher (95.92%) than the Control groups (85.02%).

**Conclusions:**

In contrast to the Control group, the Test group demonstrated superior MAGRD resolution in achieving a decrease in GRD and GRW as well as a higher increase in WKG and GT. These findings resulted into a substantially more CRC for the Test group.

**Trial registration:**

Registration no. CTRI/2022/09/045845.

Registered on: 26/09/2022

## Introduction

Essential elements of a beautiful and alluring smile are the harmonious arrangement of teeth and gingival tissue, which are suitably displayed within the parameters of the lip frame. The gingival margins and the intact interdental papillae, which together make up the pink aesthetic, represent the homogeneous blending of soft tissue over the alveolar bone and teeth [1,2]. The above characteristics represent a flawless smile which emanates confidence and well-being in an individual. Gingival recession is brought on by trauma from the occlusion, poor brushing techniques, tooth malposition, and frenum pull, which exposes the root surface [3,4]. In addition to the resultant esthetic problem, gingival recession also induces root sensitivity, cervical abrasions which act as plaque retentive areas and increase the susceptibility to root caries [5,6].

For the treatment of gingival recession problems, connective tissue grafts have become the gold standard over time. The preparation of the second surgical site, significant surgical trauma and the resulting discomfort, and the scarcity of donor tissue, which limits its use, are the procedure’s main drawbacks [7].

In order to treat multiple adjacent gingival recession defects (MAGRD), Zadeh H. redesigned the tunnel method by introducing the vestibular incision subperiosteal tunneling access (VISTA) approach [8]. In addition to ensuring a sufficient blood supply, the minimally invasive technique necessitates a tiny incision that involves completely undermining the periosteum in the root coverage area. This will help to coronal reposition the flap over the exposed root surface [9].

In order for the regenerated tissue to be sustainable over time, it is thought that the root coverage treatments should not only result in full root coverage but also in improved gingival thickness and keratinized gingiva width [10,11]. Choukroun et al. were the first to introduce platelet rich fibrin (PRF), an autologous leukocyte and platelet rich biomaterial [12]. With the emergence of newer modification in centrifugation protocols for PRF, low-speed led to the creation of A-PRF by Ghanaati S et al [13], which contained more platelets and inflammatory cells.

A-PRF membrane-based root coverage and subepithelial connective tissue graft (SCTG) were evaluated in a study by Anegundi RV et al [14,15] to treat gingival recession type 1. The study’s findings indicated that both A-PRF and SCTG may be used to treat gingival recession. The authors further claimed that A-PRF could also be considered as a viable alternative to CTG.

Since soft-tissue cone beam computed tomography (ST-CBCT) is the most accurate and non-invasive technique for determining gingival thickness and can clearly contour soft tissues in the esthetic area, it is used in MAGRD therapy to study soft tissue thickness and root coverage.

Literature on the treatment and evaluation of MAGRD using A-PRF in combination with VISTA is few. In order to assess and compare the VISTA approach with and without A-PRF in the treatment of MAGRD in the maxillary anterior region, this study was designed.

## Materials and methods

**Fig 1:**
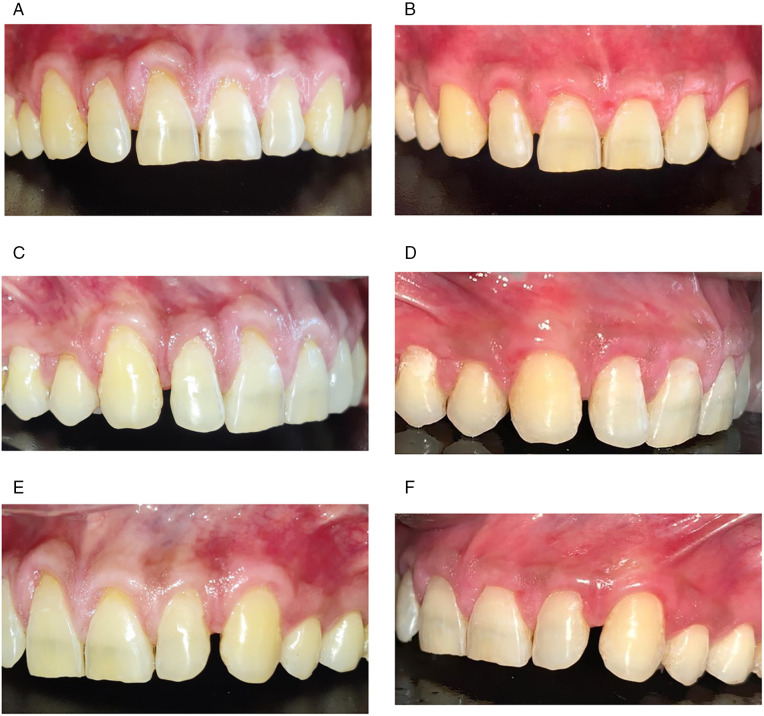
Preoperative and postoperative clinical presentation. (a) Clinical pictures MAGRD at baseline (Front View), (b) Post-operative clinical picture at 12 months (Front view), (c) MAGRD at baseline (Test group), (d) Post-operative clinical picture at 12 months (Test group), (e) MAGRD at baseline (Control group), (f) Post-operative clinical picture at 12 months (Control group).

The CONSORT criteria were followed in this investigation. The Department of Periodontics and Implant Dentistry conducted this interventional, split mouth, single masked, randomized controlled experiment from July 2022 to February 2024. For this investigation, 25 individuals with bilateral MAGRD in the maxillary anterior region, 19 of whom were male and 6 of whom were female, between the ages of 20 and 55, were included Fig 1. The study’s design and protocol were planned in accordance with the 1975 Helsinki Declaration standards, which were updated in 2000. They were then submitted to the Institutional Ethics Committee (EC/NEW/INST/2020/687) for approval. Before giving their written informed consent, all participants received verbal and written descriptions of the study’s objectives, risks, and advantages.

### Inclusion and exclusion criteria

The study included patients who met the following inclusion criteria: a) patients between the ages of 18 and 55; b) had teeth that were in good alignment; c) had full mouth bleeding score percentage (FMBS%) and full mouth plaque score percentage (FMPS%) of less than 15%; and d) had bilateral RT1 MAGRD, or Miller’s Class I & II gingival recession, with no interproximal soft and hard tissue loss. The exclusion criteria were a) Patients who were smokers, b) with a history of systemic disease, c) were pregnant or nursing mothers, d) had undergone periodontal therapy within the previous six months, e) presence of caries, deep cervical abrasion and restoration at the site gingival recession defects were excluded and e) had any other metabolic condition known to alter healing response.

### Sample size calculations

In light of a study by Hegde et al. [16] which showed that the response difference between matched pairs had a standard deviation of 1.43 and was regularly distributed. To be able to reject the null hypothesis, we would require 22 patients if the genuine difference in the mean response of matched pairs was 1.75. With a probability (power) of 95%, this response difference of 0 would be accomplished. This test had a Type I error probability of 0.05 with a 95% confidence interval. The predicted sample size was 25 patients, assuming a 10% dropout rate.

A computer-generated random number table was utilized to assign the sites to the treatment groups.

### Pre-surgical measurements

On the day of the procedure, the same examiner (RK) took clinical data from each patient utilizing bespoke stents and a UNC-15 periodontal probe before the surgery. FMPS% and FMBS% were measured at baseline, six and twelve months following surgery in order to assess gingival health and oral hygiene. The parameters recorded were as follows:

1. **Gingival recession depth (GRD):** The distance between the gingival edge and the most apical point of the CEJ.
2. **Gingival recession width (GRW):** This was determined by measuring the distance on a horizontal line tangential at the CEJ between the tooth’s mesial and distal gingival margins.
3. **Probing depth (PD):** This was determined by measuring the distance between the gingival margin and the gingival sulcus bottom.
4. **Clinical attachment level (CAL):** The distance between the bottom of the sulcus and the CEJ was used to measure it.
5. **The width of keratinized gingiva (WKG):** was calculated by measuring the distance between the gingival edge and the mucogingival junction (MGJ). The position of the MGJ was ascertained visually.
6. **Gingival thickness (GT):** Radiographic soft tissue assessment of GT was done from the margin of gingiva at 2 mm, 4 mm and 6 mm apical to the margin on CBCT scans.
7. **Complete root coverage (CRC)**: The value was evaluated using the formula

$$\frac{\text{Preoperative}\;\text{recession}\;\text{depth}\;-\;\text{Postoperative}\;\text{recession}\;\text{depth}}{\text{Preoperative}\;\text{recession}\;\text{depth}}\;\times \text{100}.$$

Using the formula provided by Shieh AT et al, the CRC was assessed after therapy [17].

#### Preparation of composite buttons on buccal surface of the teeth.

For the coronal advancement of gingival margin composite buttons were prepared on the mid-buccal region of every tooth with recession with the help of orthodontic separator bands.

### Surgical procedure

Each patient was treated with VISTA method alone in the Control group and VISTA with A-PRF in the Test group. Prior to the therapy, the injured root surface was bio modified by using 24% EDTA for two minutes to increase coagulum adhesion to the root surface and remove the smear layer from the dentin tubule. In this investigation, the surgical protocol for Zadeh H.‘s [8] VISTA surgical approach was adhered to (Fig 1a–1f).

**Fig 2:**
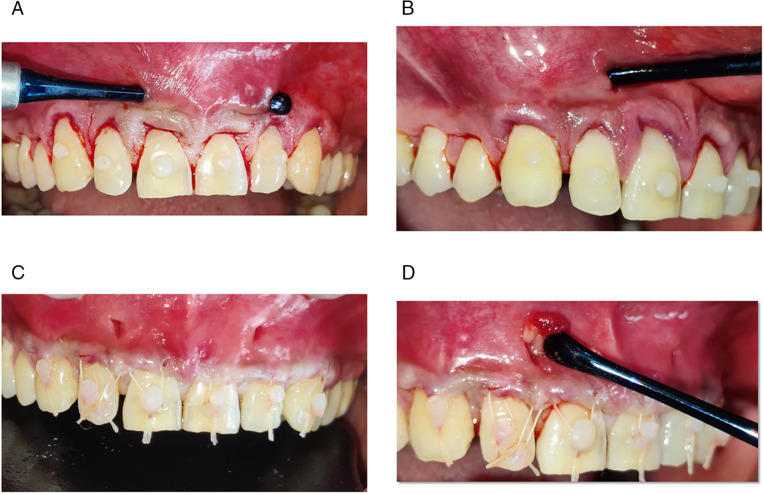
Intraoperative clinical presentation. (a) Tunnel preparation (Control group), (b) Tunnel preparation (Test group), (c) Coronal advancement of tunnel using composite buttons. (Control group), (d) Coronal advancement of tunnel using composite buttons along with A-PRF membrane placement (Test group).

In order to seal the knots of anchoring sutures and prevent the tissue apically from relapsing during the early stages of healing, a tiny amount of flowable composite resin was applied to the prepared buccal face of each tooth and light-cured (Fig 2a–2d).

The Test group underwent a similar surgical technique, but with the addition of inserting the A-PRF membrane, which was done through a tiny vertical incision and placed inside the tunnel, covering the whole recession area.

A-PRF was made in accordance with Ghanaati et al.‘s protocol [13]. Venous blood was collected without anticoagulant in 10 mL sterile glass tubes and immediately centrifuged at 1500 rpm for 14 minutes using REMI centrifuge (R-8C REMI Elektrotechnik Ltd. India). The fibrin clot was separated from the red blood cell layer and gently compressed between sterile gauze to form membranes, which were then inserted into the tunnel to cover the recession defects.

### Post-surgical management

**Fig 3:**
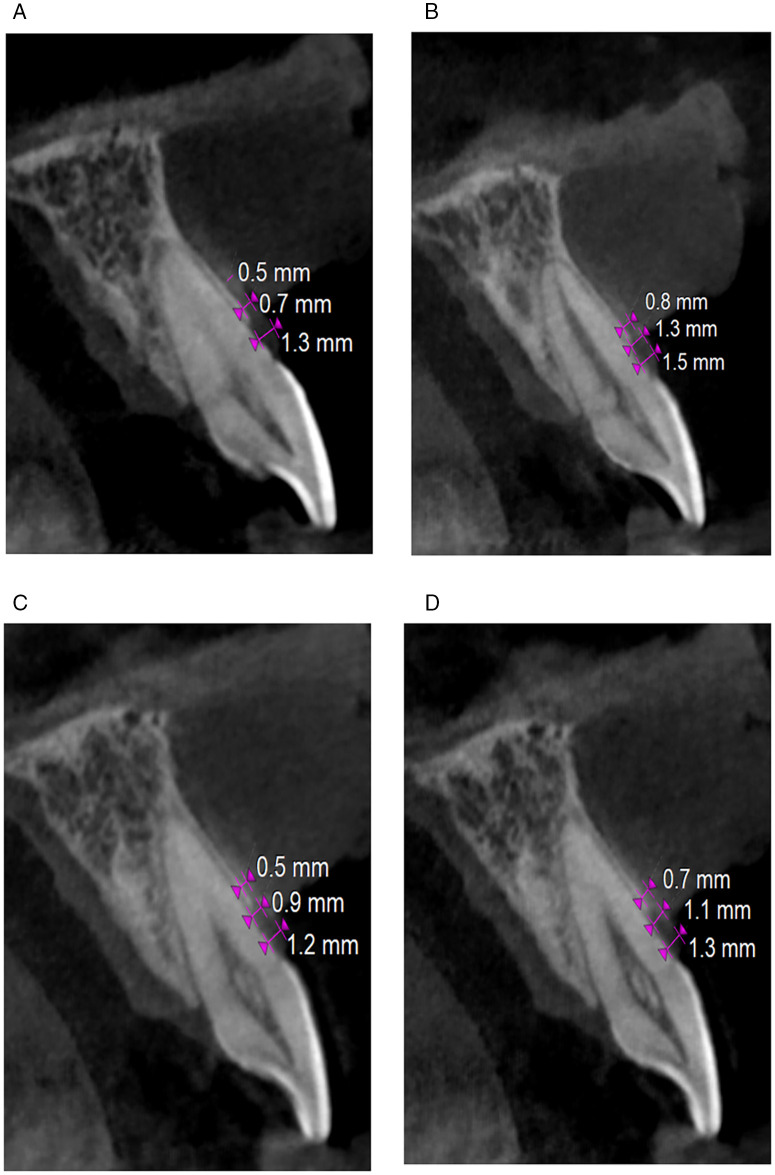
Comparison of pre-operative and 12 months ST-CBCT images of Control Group and Test Group. (a) Pre-operative ST-CBCT findings (Test group), (b) Post-operative ST-CBCT findings at 12-months (Test group), (c) Pre-operative ST-CBCT findings (Control group), (d) Post-operative ST-CBCT findings at 12-months (Control group).

Antibiotics and analgesics were administered to each patient. Oral prophylaxis was performed and the oral hygiene guidelines were re-instituted during the follow-up. While the ST-CBCT measures of the GT were performed at baseline and 12 months post-operatively, clinical measurements were taken at baseline, 6 months, and 12 months following surgery (Fig 3a–3d).

The CBCT scans were acquired with a Carestream CS 9300 unit (Carestream Dental, USA) at a voxel size of 0.18 mm, with standardized head positioning and field of view. Images were analyzed using CS 3D Imaging software (v3.10). All measurements were performed by a single calibrated examiner, with intra-examiner reliability assessed using intra-class correlation coefficients (ICC = 0.91). Inter-examiner reliability was also evaluated (ICC = 0.89).

### Statistical analysis

All statistical analyses were conducted using IBM SPSS version 21.0 (IBM Corp., USA), and statistical significance was tested at a 5% level. While the categorical component gender was represented in terms of frequency and percentage, the demographic parameter age was reported in terms of mean and standard deviation. The repeated measures analysis of variance (ANOVA) test and the post hoc Bonferroni test were used to compare the parameters, including PD, GRD, CAL, WKG, and GRW, at various time points within the group. The mean and standard deviation were used to express each of the quantifiable clinical factors. The paired t-test was used to compare the parameters of the Control (VISTA alone) and Test (VISTA + A-PRF) groups in light of the split mouth study design. The paired t-test was used to compare the GT at 2 mm, 4 mm, and 6 mm between baseline and 12 months within the groups. The unpaired t-test was used to compare the change in GT between the Control and Test groups at 2 mm, 4 mm, and 6 mm from baseline to 12 months.

## Results and discussion

A total of 25 patients exhibiting bilateral 216 MAGRD were equally allotted in the Control and Test groups. The satisfactory oral hygiene maintenance was reflected in the values of FMPS% and FMBS% which were significantly reduced in both the test and the control groups from baseline to 12 months post therapy. A clear trend emerged in both groups exhibited significant improvements in GRD, GRW, CAL, and WKG from baseline to 12 months, with the Test group consistently outperforming the Control group. In particular, the Test group achieved greater reductions in gingival recession depth and width, accompanied by superior gains in width of keratinized gingiva and gingival thickness. These outcomes translated into a markedly higher percentage of complete root coverage in the Test group, emphasizing the clinical benefit of incorporating A-PRF into the VISTA technique.

The parameters when compared at different time points, i.e., baseline, 6- and 12-months observation period within the group using repeated measures analysis of variance (ANOVA) test were found to be statistically significant Table 1.

**Table 1:** Mean difference between different time points for all parameters in Control and Test Group.

Parameters	Groups	(Mean±SD) at time points			F Value	Significance
		Baseline	6 Months	12 Months		
PD(mm)	Control	0.92 ± 0.31	0.16 ± 0.24	0.04 ± 0.11	430.28	0.0001*
	Test	0.81 ± 0.30	0.13 ± 0.24	0.02 ± 0.07	399.554	0.0001*
CAL(mm)	Control	4.5 ± 0.53	1.04 ± 0.53	0.22 ± 0.33	3347.64	0.0001*
	Test	4.71 ± 0.65	0.86 ± 0.44	0.1 ± 0.21	2485.52	0.0001*
WKG(mm)	Control	3.42 ± 0.90	3.97 ± 0.82	4.53 ± 0.91	109.51	0.0001*
	Test	3.51 ± 0.91	4.18 ± 0.81	5 ± 0.86	209.62	0.0001*
GRD(mm)	Control	3.59 ± 0.44	0.88 ± 0.40	0.18 ± 0.31	2966.96	0.0001*
	Test	3.89 ± 0.56	0.74 ± 0.33	0.08 ± 0.18	2402.72	0.0001*
GRW(mm)	Control	4.41 ± 0.71	3.24 ± 0.65	2.13 ± 0.59	510.44	0.0001*
	Test	4.44 ± 0.65	3.17 ± 0.53	1.67 ± 0.74	546.08	0.0001*

PD- probing depth, CAL- clinical attachment level, WKG- width of keratinized gingiva, GRD- gingival recession depth, GRW- gingival recession width, mm-millimeter, SD-standard deviation, F value- denotes use of analysis of variance (ANOVA).

The findings reflected in the CRC which was observed to be 95.92% in Test group when compared to Control group where it was 85.02% (Table 2).

**Table 2:** Mean difference between Control and Test group for different parameters.

Parameters	Time points	Groups	Mean±SD	Mean difference	T-value	Significance
**PD (mm)**	Baseline	Control	0.92 ± 0.31	0.11	2.572	0.011
		Test	0.81 ± 0.30			
	6 Months	Control	0.16 ± 0.24	0.04	1.105	0.27
		Test	0.13 ± 0.24			
	12 Months	Control	0.04 ± 0.11	0.02	1.516	0.131
		Test	0.02 ± 0.07			
**CAL(mm)**	Baseline	Control	4.5 ± 0.53	0.21	2.557	0.011
		Test	4.71 ± 0.65			
	6 Months	Control	1.04 ± 0.53	0.18	2.749	0.006*
		Test	0.86 ± 0.44			
	12 Months	Control	0.22 ± 0.33	0.12	3.184	0.002
		Test	0.1 ± 0.21			
**WKG (mm)**	Baseline	Control	3.42 ± 0.90	0.09	0.713	0.477
		Test	3.51 ± 0.91			
	6 Months	Control	3.97 ± 0.82	0.21	1.874	0.062
		Test	4.18 ± 0.81			
	12 Months	Control	4.53 ± 0.91	0.47	3.924	0.0001*
		Test	5 ± 0.86			
**GRD (mm)**	Baseline	Control	3.59 ± 0.44	0.3	4.455	0
		Test	3.89 ± 0.56			
	6 Months	Control	0.88 ± 0.40	0.14	2.802	0.006*
		Test	0.74 ± 0.33			
	12 Months	Control	0.18 ± 0.31	0.1	2.799	0.006*
		Test	0.08 ± 0.18			
**GRW (mm)**	Baseline	Control	4.41 ± 0.71	0.04	0.401	0.689
		Test	4.44 ± 0.65			
	6 Months	Control	3.24 ± 0.65	0.07	0.857	0.392
		Test	3.17 ± 0.53			
	12 Months	Control	2.13 ± 0.59	0.45	4.974	0.0001*
		Test	1.67 ± 0.74			
**%RC**	At 12 Months	Control	95.37	1.74	1.69	0.091
		Test	97.1			

PD- probing depth, CAL- clinical attachment level, WKG- width of keratinized gingiva, GRD- gingival recession depth, GRW- gingival recession width, mm-millimeter, SD-standard deviation, RC- root coverage, t value- denotes use of t-test.

Tables 3 and 4 signify the comparison of radiographic measurement changes in GT from baseline to 12 months at 2 mm, 4 mm and 6 mm between Control and Test group performed using unpaired t-test.

**Table 3:** Mean difference between different time points for GT at 2 mm, 4 mm and 6 mm in Control and Test Group.

Parameters	Groups	Time points	Mean±SD	Mean difference	T value	Significance
**GT at 2 mm**	Control	Baseline	1.29 ± 0.22	0.79	25.734	0.0001*
		12 Months	2.08 ± 0.20			
	Test	Baseline	1.29 ± 0.21	0.96	31.139	0.0001*
		12 Months	2.25 ± 0.22			
**GT at 4 mm**	Control	Baseline	1.28 ± 0.24	0.8	32.468	0.0001*
		12 Months	2.09 ± 0.16			
	Test	Baseline	1.2 ± 0.17	1.03	40.515	0.0001*
		12 Months	2.23 ± 0.20			
**GT at 6 mm**	Control	Baseline	1.2 ± 0.21	0.87	31.322	0.0001*
		12 Months	2.08 ± 0.21			
	Test	Baseline	1.32 ± 1.16	0.95	8.435	0.0001*
		12 Months	2.27 ± 0.20			

GT-gingival thickness, mm-millimeter, SD- standard deviation, T- denotes use of t test.

**Table 4:** Mean difference between Control and Test group for GT at 2 mm,4 mm and 6 mm.

Time Points	Levels	Groups	Mean±SD	Mean difference	T-value	Significance
**Baseline**	At 2 mm	Control	1.29 ± 0.22	0	0.063	0.95
		Test	1.29 ± 0.21			
	At 4 mm	Control	1.28 ± 0.24	0.07	2.614	0.010*
		Test	1.2 ± 0.17			
	At 6 mm	Control	1.2 ± 0.21	0.12	1.072	0.285
		Test	1.32 ± 1.16			
**12 Months**	At 2 mm	Control	2.08 ± 0.20	0.16	5.755	0.0001*
		Test	2.25 ± 0.22			
	At 4 mm	Control	2.09 ± 0.16	0.14	5.93	0.0001*
		Test	2.23 ± 0.20			
	At 6 mm	Control	2.08 ± 0.21	0.19	7.101	0.0001*
		Test	2.27 ± 0.20			

mm-millimeter, SD- standard deviation, T-denotes use of t test.

The goal of the current split-mouth randomized controlled clinical trial was to evaluate and contrast the clinical effectiveness of the VISTA approach in treating MAGRD with and without A-PRF. The issue is more severe in MAGRD, nevertheless, because of the extent of the avascular recipient area, which frequently makes it challenging to restore the blood flow required for the grafted tissue to heal. Aside from this, gingival recession may recur or insufficient root covering may result from muscle contraction during healing [9]. When considering the majority of these characteristics, the VISTA treatment is considered a dependable method for correcting such problems because it is minimally invasive, does not impact the blood supply, causes minimal surgical trauma, and nevertheless improves all clinical measures [18].

The comparative analysis in Tables 2–4 demonstrates that, although minor baseline differences were observed between the Control and Test groups, the split-mouth randomized design ensured that each patient served as their own control, thereby minimizing inter-subject variability. Consequently, the changes observed over time within each group provide the most reliable measure of treatment efficacy. Both groups showed statistically significant improvements in clinical parameters; however, the Test group consistently achieved greater reductions in GRD and GRW, higher gains in WKG, and superior increases in gingival thickness at all measured levels. These advantages translated into a higher percentage of complete root coverage at 12 months. Taken together, the findings support the conclusion that the incorporation of A-PRF into the VISTA technique yields more favorable clinical and radiographic outcomes than VISTA alone, despite baseline variations.

Given that the differences between GRD and GRW are clinically apparent and valued by the patients, it appears that the parameters have real advantages for the patients. These results are consistent with those of Rajendran et al [19]. who compared minimally invasive coronally advanced flap (Test) with modified coronally advanced flap (Control). The GRD reduction from baseline to 6 months was 2.22 mm and 3.05 mm for the Control and Test groups. The observations in our study are similar but the results achieved as far as GRD reduction were 3.41 mm and 3.81 mm for the Control and Test groups which were better than the said trial. The observations for GRW reductions indicated a reduction of 3.1 mm and 3.05 mm in the above study while in the present study the differences were 2.28 mm and 2.78 mm for Control and Test groups respectively. Twenty pinhole surgical procedures with and without a PRF were compared in a study by Trivedi et al [20]. to treat gingival recession. The GRW values showed 2.47 mm and 2.51 mm reductions at 12 months after therapy, although the GRD reductions were slightly smaller. The variations in results can be attributed largely to the differences in the treating procedures, period of observation and also the baseline defect morphology which could have been somewhat dissimilar. Incorporation of A-PRF significantly enhanced regenerative outcomes as compared to sites where the biomaterial is not used [21,22]. In the present study suspended sutures were attached to the composite buttons and were covered with flowable composite for a period of two weeks post treatment so that there is no alteration in the marginal position during the early healing period.

Trivedi et al reported a considerable increase of 1.49 mm and 0.79 mm of WKG in both the groups respectively which are similar to those reported by Chao [23]. who obtained an increase of 1.3 mm and these results have been substantiated by the present study wherein the increase in WKG was 1.49 mm and 1.11 mm for the respective groups. Such an increase in WKG is ascribed to the placement of A-PRF which has been credited to change the thin gingival biotype into a thick biotype [24].

In the present study the difference between mean values of GT which was measured with ST-CBCT scans at 2, 4 and 6 mm apical to the gingival margin was found to be significant from baseline to 12 months in both the groups but was seen to be greater for the Test group. The maximum change occurred at 4 mm apical to the gingival margin which was 1.03 mm, supposedly due to the fact that it is the region where the capillaries would have their respective terminal endings making it an ideal area for regeneration of the tissue due to greater nutritional supply. These findings are similar to those reported previously [23,25]. The reports by Dandu et al mentioned an increase which was a bit less with GT of 0.45 mm at 18 months time interval [26] However, the surgical procedure used was coronally advanced flap with a different biomaterial. Variation in results can also be obtained owing to the differences of preexisting biotype.

In the present study mean root coverage at 12 months was 97.10% and 95.36% in Test and Control groups. In Test group 88 out of 109 (95.92%) sites showed CRC while 78 out of 109 sites (85.02%) showed CRC in Control group. Similar results were obtained in the study conducted by Rajeshwari SR. et al [27] in which VISTA +PRF group showed 93.95% whereas Zucchelli’s technique + PRF revealed 96.84% of CRC. Another study by Hegde S et al [16] achieved a bit lesser CRC in their investigation which can be due to the discrepancies in the pre-existing morphology of the tissues and defects. Additionally, the coronal repositioning achieved at the time of suturing and maintenance of it in the same position in the early healing phases also is known to influence the results.

There were some limitations of the study including by the absence of histologic evaluation of the exact nature of attachment achieved and a small sample size. In view of the ever-increasing demand of minimally invasive procedures, further investigations on a larger sample size and a greater observation period are desired. While the split-mouth randomized design minimized inter-patient variability and enhanced internal validity, the sample size of 25 patients may limit the generalizability of these findings. Larger, multicenter clinical trials involving more diverse populations are needed to confirm the external validity and broader applicability of our results. Furthermore, although some baseline differences in parameters between groups were noted, statistical analyses were adjusted to account for these variations, and conclusions were primarily drawn from longitudinal changes within each group rather than isolated cross-sectional differences.

## Conclusions

Overall, the findings led to CRC of 95.92% in the Test group which was significantly greater than the Control group. A-PRF as a biomaterial has proved to have distinct benefits which are emphasized and observed through the results of this study.

## Supporting information

S1 File Primary data file. (XLSX)

S2 File CONSORT Checklist. (DOC)

S3 File Study protocol. (PDF)

S1 Table Supplementary tables. (DOCX)
