# Correction: Effectiveness of vestibular incision subperiosteal tunnel access (VISTA) technique with or without A-PRF in treatment of multiple adjacent gingival recession defects (MAGRD): A 12 months CBCT study

**DOI:** 10.1371/journal.pone.0348892

**Published:** 2026-05-05

**Authors:** Prabhnoor Tuli, Abhay P. Kolte, Rajashri A. Kolte, Vrushali N. Lathiya, Vinisha A. Bajaj, Shahabe Saquib Abullais, Manea M. Alahmari

The Funding statement for this article is incorrect. The correct Funding statement is as follows: The authors extend their appreciation to the Deanship of Scientific Research and graduate studies at King Khalid University for supporting this work through Large group Project RGP-2/123/46.
