# Supplementary material for: Effectiveness of vestibular incision subperiosteal tunnel access (VISTA) technique with or without A-PRF in treatment of multiple adjacent gingival recession defects (MAGRD): A 12 months CBCT study
Source: PLoS One. 2025 Dec 23;20(12):e0338823. doi: 10.1371/journal.pone.0338823 (PMC12725620; doi:10.1371/journal.pone.0338823)
Supplement: S3 File — (PDF) [file pone.0338823.s003.pdf]

TITLE:

Evaluation & Correlation of facial and anterior teeth proportions amongst different facial types.

INTRODUCTION:

Patients seeking restorative and periodontal treatment prioritise esthetics above anything else. In esthetic restorations, facial landmarks are critical for achieving a pleasing facial harmony expression. The advancement of new dental materials and techniques has resulted in a broader variety of treatment alternatives that increase the chances of a pleasing result. For esthetics assessment, cosmetic dentistry, and reconstructive operations, a facial evaluation has become a necessary component. The human face has a variety of shapes determined by the facial index that is calculated using facial height by the bizygomatic width of the patients that classifies the facial forms as broad average and narrow. The size, shape, and arrangement of the maxillary anterior teeth are the most important aspects in achieving a harmonious anterior dentition. The maxillary anterior teeth must be proportioned to the face morphology to appear appealing. The Golden proportion that is calculated by the ratio of the individual width of a tooth (central incisor , lateral incisor , canine) by the total width of all maxillary anterior teeth multiplied by 100. Several studies prove that Golden Proportion is a useful tool for evaluating symmetry, dominance, and proportion in assessing tooth arrangement and performing esthetic dental therapy. Several studies indicate the use of anatomic measurements, such as the intercomissural width, facial index to aid in restoring and establishing the correct size of the anterior teeth. Hence the aim of the present study is to Evaluate & Correlate facial and tooth proportions amongst different facial types in periodontally healthy patients.

PRIMARY RESEARCH QUESTION:

Is there any correlation of facial and maxillary anterior teeth proportions amongst different facial types in periodontally healthy patients.

NULLS HYPOTHESIS:

There is no correlation of facial and maxillary anterior teeth proportions amongst different facial types in periodontally healthy patients.

PRIMARY HYPOTHESIS:

There is a significant correlation of facial and maxillary anterior teeth proportions amongst different facial types in periodontally healthy patients.

REVIEW OF LITERATURE:

1) **Asikul Wadud** et.al 2021 stated that no GP or GS was found compared with the 6 maxillary anterior teeth in the studied population. Anterior teeth measurements with different facial landmarks showed a significant correlation. The W/H ratio of central incisor, lateral incisor, and canine showed no significant difference with 3 face types except W/H ratios between average and narrow face types ($p<.05$). The size and shape of the maxillary anterior teeth were the most important factors to achieve pleasing dental and facial esthetics. The authors concluded that the data obtained from this study may help to provide guidelines for prosthetic management with proper esthetic outcomes in Thai populations according to their facial proportions
2) **Hasanreisoglu U, Berksun S, Aras K, Arslan I** have found proportional smile evaluations are generally based on the perceived size of the anterior teeth from the frontal view. In this respect, the central incisors are the most dominant anterior teeth in the dental arch because they can be seen in their full size and linear measurements such as facial index, interalar distance to intercanine distance which might be used as one of the initial references in establishing the width of the central incisors or the localization of the canines, particularly for women.
3) **Mishra M** et al. 2016 has found strong correlation between interpupillary distance, inter-alar width & combined width of maxillary anterior teeth in specific racial populations. It was concluded that inter-alar width, interpupillary distance & intercommisural width in Aryans and interpupillary distance in Mongoloids can be used to determine the combined width of maxillary anterior teeth

PRIMARY OBJECTIVE:

Evaluation & correlation of facial and maxillary anterior teeth proportions amongst different facial types in periodontally healthy patients.

SECONDARY OBJECTIVES:

1) To evaluate facial height and facial width in the study population.
2) To evaluate facial index using the facial height by its facial width in percentage and categorize the facial type as broad, average, or narrow.
3) To evaluate the width and height of maxillary anterior teeth from central incisors to canine on both sides.
4) To evaluate the intercommisural width in study population.
5) To correlate the facial height, facial width, facial type with the maxillary anterior tooth proportions and intercommisural width.

RESEARCH METHODOLOGY:

SAMPLE SIZE:

Total of 200 patients visiting to the Department of Periodontics and Implantology at VSPM Dental College and Research Centre, Nagpur will be selected and assessed clinically.

STUDY DESIGN:

Cross-sectional study.

STUDY SETTING:

It will be conducted in Department of Periodontics and Implantology at VSPM Dental College& Research Centre, Nagpur and will be initiated after clearance from the Institutional Ethics Committee of VSPM Dental College & Research Centre, Nagpur.

Patients will be divided into two groups according to gender as follows:

Group I – 100 periodontally healthy males in the age range of 20-40 years.

Group II - 100 periodontally healthy females in the age range of 20-40 years.

STUDY POPULATION:

Patients visiting in Department of Periodontics and Implantology as per inclusion criteria will be included in the study groups.

INCLUSION CRITERIA:

1. Patients who are willing to participate and sign the informed consent for this clinical trial.
2. Males and females between the age group of 20-40 years.
3. Healthy gingiva as assessed with a Plaque Index (Silness & Loe 1964) and Gingival Index (Loe & Silness 1963) between the score of 0–1.
4. Patients with fully erupted well-aligned maxillary Central Incisors, Lateral Incisors and Canines without any orthodontic anomaly.

EXCLUSION CRITERIA:

1. Artificial crowns, fillings, attrition on anterior teeth.
2. Crowding or spacing in the anterior teeth.
3. Evidence of incomplete passive eruption.
4. Translocated/Tilted teeth.
5. Pregnancy or a history of taking medications known to increase the risk of gingival hyperplasia.
6. Patients diagnosed with destructive periodontal disease.
7. Facial asymmetry.

MATERIALS AND METHOD:

Total of 200 participants, comprising 100 males and 100 females within the age range of 20 to 40 years, as per the inclusion criteria will participate in the study. The participants will be divided in two groups.

Group I – 100 males

Group II– 100 females

The study will be initiated after clearance from the Institutional Ethics Committee of VSPM Dental College & Research Centre, Nagpur. The study parameters of the patients will be assessed clinically and on digital photographs. Frontal digital photographs and retracted photographs of the patients will be taken while for assessment of facial proportions with proportions of maxillary anterior teeth and intercommisural width in patients.

Determination of Facial height (FH) in mm:

For the measurement of FH, FH will be measured from trichion (the front line of the hair in the midline) to menton (the lowest soft tissue point of the mandible) at rest.

Determination of facial width (FW) in mm:

For the measurement of facial width the maximum horizontal distance between right and left facial boundary [bizygomatic width] at rest will be measured.

Determination of teeth width (TW) in mm:

The teeth width dimension will be obtained by measuring the maximum distance between the mesial and distal contact points of the tooth using digital caliper and using the caliberation on the DTS PRO software.

Determination of teeth height (TH) in mm:

The height dimension will be obtained by measuring the maximum vertical distance from the cervical margin to the incisal edge of the tooth using digital caliper and the calibrations on the DTS PRO software.

Determination of intercommisural width(ICOW) in mm.

The ICOW will be determined by measuring the maxillary lip vermilion from the right commissure to the left commissure with digital DTS PRO software.

STATISTICAL ANALYSIS:

The data will be statistically analysed after completion of the study.

INSTITUTION NEEDED FOR STUDY:

VSPM Dental College & Research Centre, DigdohHills, HingnaRoad, Nagpur.

STUDY DURATION:3 months.

APPROXIMATE COST: Rs. 15000/-

REFERENCES:

1) **Hasanreisoglu U, Berksun S, Aras K, Arslan I.** An analysis of maxillary anterior teeth: facial and dental proportions. J Prosthet Dent. 2005 Dec;94(6):530-8. doi: 10.1016/j.prosdent.2005.10.007. PMID: 16316799.
2) **Asikul Wadud. Face Proportions, and Analysis of Maxillary Anterior Teeth and Facial Proportions in a Thai Population in the Department of Prosthodontics, Update Dental College and Hospital, Aichi Nagar, Turag, Dhaka 1711, Bangladesh; TODENTJ-15-398.**
3) **Mishra MK, Singh RK, Suwal P, Parajuli PK, Shrestha P, Baral** D. A comparative study to find out the relationship between the inner inter-canthal distance, interpupillary distance, inter-commissural width, inter-alar width, and the width of maxillary anterior teeth in Aryans and Mongoloids. ClinCosmetInvestig Dent. 2016; 22:8:29-34.
4) **Condon M, Bready M, Quinn F, O'Connell BC, Houston FJ, O'Sullivan M.** Maxillary anterior tooth dimensions and proportions in an Irish young adult population. J Oral Rehabil. 2011;38(7):501-8.
5) **Deogade SC, Mantri SS, Saxena S, Daryani H.** Correlation between combined width of maxillary anterior teeth, interpupillary distance and intercommissural width in a group of Indian people. Int J Prosthodont Restor Dent. 2014;4(4):105-11.
6) **Petekkaya, Emine & OzandaçPolat, Sema & Kabakçı et al.** Evaluation of Dental Golden Ratio in Terms of Facial Esthetics. J Ind Orth Soc. 2021;55:285-290.

INFORMED CONSENT

Evaluation & correlation of facial and anterior teeth proportions amongst different facial types.

NAME OF PRINCIPLE INVESTIGATOR: DR. ABHAY KOLTE

NAME OF CO-INVESTIGATOR : DR. PRABHNOOR TULI

I confirm that I understand the subject information for the above study and have had the opportunity to ask questions which have been answered fully. I understand that my participation is voluntary and I am free to withdraw at any time, without giving any reason, without my medical care or legal rights being affected. I understand that sections of any medical notes may be looked at by responsible individuals where it is relevant to my taking part in this research.

I give permission for these individuals to access my records that are relevant to this research.

I agree to take part in the above study.

NAME OF THE PARTICIPANT **SIGNATURE** **DATE**

VSPM's DENTAL COLLEGE AND RESEARCH CENTRE, NAGPUR

DEPARTMENT OF PERIODONTOLOGY

SHORT STUDY CASE HISTORY PROFORMA

Evaluation & Correlation of facial and anterior teeth proportions amongst different facial types.

**NAME -**	**OPD NO. -**
**AGE/SEX -**	**DATE -**
**ADDRESS -**	**MOBILE NO -**

CHIEF COMPLAINT:

PAST DENTAL HISTORY:

PAST MEDICAL HISTORY:

ORAL HYGIENE HABIT:

TEETH PRESENT:

Parameters measured on frontal photographs (in mm)	
Facial height (FH) measured on frontal photographs	
Facial width (FW) measured on frontal photographs	
Facial index	

Facial type	Tooth type
Broad	
Average	
Narrow	

Intercommisural width (ICW) measured on frontal photographs	

Height of the anterior maxillary tooth measured on photograph<br>The height dimension will be obtained by measuring the maximum vertical distance from the cervical margin to the incisal edge of the tooth						
Tooth No	13	12	11	21	22	23
In mm						

Width of the maxillary anterior tooth measured on photograph<br>The width dimension will be obtained by measuring the maximum distance between the mesial and distal contact points of the tooth						
Tooth no	13	12	11	21	22	23
In mm						
