# Supplementary material for: Effectiveness of vestibular incision subperiosteal tunnel access (VISTA) technique with or without A-PRF in treatment of multiple adjacent gingival recession defects (MAGRD): A 12 months CBCT study
Source: PLoS One. 2025 Dec 23;20(12):e0338823. doi: 10.1371/journal.pone.0338823 (PMC12725620; doi:10.1371/journal.pone.0338823)
Supplement: S1 Table — (DOCX) [file pone.0338823.s004.docx]

**TABLES**

Table 1: Mean values of the parameters at baseline, 3 months, and 6 months in test group

| **Parameters** | **Time point** | **N** | **Minimum** | **Maximum** | **Mean** | **Std. Deviation** |
| --- | --- | --- | --- | --- | --- | --- |
| Periodontal probing depth | Baseline | 108 | .00 | 1.00 | 0.81 | 0.30 |
|  | 3months | 108 | .00 | 1.00 | 0.13 | 0.24 |
|  | 6months | 108 | .00 | .40 | 0.02 | 0.07 |
| Recession depth | Baseline | 108 | 2.50 | 5.50 | 3.89 | 0.56 |
|  | 3months | 108 | .00 | 1.50 | 0.74 | 0.33 |
|  | 6months | 108 | .00 | .50 | 0.08 | 0.18 |
| Percentage root coverage | 6months | 108 | 75.00 | 100 | 97.10 | 6.81 |
| Clinical attachment level | Baseline | 108 | 3.00 | 6.50 | 4.71 | 0.65 |
|  | 3months | 108 | .00 | 2.00 | 0.86 | 0.44 |
|  | 6months | 108 | .00 | .90 | 0.10 | 0.21 |
| Width of keratinized gingiva | Baseline | 108 | 2.00 | 6.00 | 3.51 | 0.91 |
|  | 3months | 108 | 3.00 | 6.50 | 4.18 | 0.81 |
|  | 6months | 108 | 3.00 | 7.00 | 5.00 | 0.86 |
| Recession width | Baseline | 108 | 3.00 | 6.00 | 4.44 | 0.65 |
|  | 3months | 108 | 2.00 | 4.00 | 3.17 | 0.53 |
|  | 6months | 108 | .00 | 3.00 | 1.67 | 0.74 |
| Gingival thickness at Baseline | At 2mm | 108 | .60 | 1.60 | 1.29 | 0.21 |
|  | At 4mm | 108 | .80 | 1.60 | 1.20 | 0.17 |
|  | At 6mm | 108 | .80 | 13.00 | 1.32 | 1.16 |
| Gingival thickness at 6 months | At 2mm | 108 | 1.80 | 2.70 | 2.25 | 0.22 |
|  | At 4mm | 108 | 1.80 | 2.60 | 2.23 | 0.20 |
|  | At 6mm | 108 | 1.90 | 2.80 | 2.27 | 0.20 |

Table 2: Mean values of the parameters at baseline, 3 months, and 6 months in control group

| **Parameters** | **Time point** | **N** | **Minimum** | **Maximum** | **Mean** | **Std. Deviation** |
| --- | --- | --- | --- | --- | --- | --- |
| Periodontal probing depth | Baseline | 108 | .00 | 1.50 | 0.92 | 0.31 |
|  | 3months | 108 | .00 | 1.00 | 0.16 | 0.24 |
|  | 6months | 108 | .00 | .50 | 0.04 | 0.11 |
| Recession depth | Baseline | 108 | 3.00 | 4.50 | 3.59 | 0.44 |
|  | 3months | 108 | .00 | 2.00 | 0.88 | 0.40 |
|  | 6months | 108 | .00 | 1.10 | 0.18 | 0.31 |
| Percentage root coverage | 6months | 108 | 71.4 | 100 | 95.37 | 8.18 |
| Clinical attachment level | Baseline | 108 | 3.00 | 5.70 | 4.50 | 0.53 |
|  | 3months | 108 | .00 | 3.00 | 1.04 | 0.53 |
|  | 6months | 108 | .00 | 1.20 | 0.22 | 0.33 |
| Width of keratinized gingiva | Baseline | 108 | 2.00 | 5.50 | 3.42 | 0.90 |
|  | 3months | 108 | 2.00 | 6.00 | 3.97 | 0.82 |
|  | 6months | 108 | 2.00 | 7.00 | 4.53 | 0.91 |
| Recession width | Baseline | 108 | 3.00 | 6.00 | 4.41 | 0.71 |
|  | 3months | 108 | 2.00 | 5.00 | 3.24 | 0.65 |
|  | 6months | 108 | 1.00 | 3.00 | 2.13 | 0.59 |
| Gingival thickness at Baseline | At 2mm | 108 | .80 | 2.00 | 1.29 | 0.22 |
|  | At 4mm | 108 | 1.00 | 3.00 | 1.28 | 0.24 |
|  | At 6mm | 108 | .80 | 1.80 | 1.20 | 0.21 |
| Gingival thickness at 6 months | At 2mm | 108 | 1.10 | 2.60 | 2.08 | 0.20 |
|  | At 4mm | 108 | 1.80 | 2.50 | 2.09 | 0.16 |
|  | At 6mm | 108 | 1.30 | 2.50 | 2.08 | 0.21 |

Table 3: Mean difference between different time points for periodontal probing depth in test group

| **Parameters** | **Time point** | **Mean** | **F value** | **Significance (p)** |
| --- | --- | --- | --- | --- |
| Periodontal probing depth | Baseline | 0.81 | 399.554 | **0.0001*** |
|  | 3months | 0.13 |  |  |
|  | 6months | 0.02 |  |  |

*Significance at p<0.05

A significant difference is present between different time points for periodontal probing depth in test group. The mean probing pocket depth is more at baseline followed by 3 months and least at 6 months.

Table 4: Post hoc Bonferroni test representing mean difference different time points for periodontal probing depth in test group

| **Time points** | | **Mean Difference** | **Significance**  **(p)** |
| --- | --- | --- | --- |
| Baseline | 3months | 0.69 | **0.0001*** |
|  | 6months | 0.79 | **0.0001*** |
| 3months | 6months | 0.11 | **0.0001*** |

*Significance at p<0.05

A significant difference is present in test group for probing pocket depth between:

Baseline and 3 months, baseline and 6 months, 3 months and 6 months.

Table 5: Mean difference between different time points for recession depth in test group

| **Parameters** | **Time point** | **Mean** | **F value** | **Significance (p)** |
| --- | --- | --- | --- | --- |
| Recession depth | Baseline | 3.89 | 2402.72 | **0.0001*** |
|  | 3months | 0.74 |  |  |
|  | 6months | 0.08 |  |  |

*Significance at p<0.05

A significant difference is present between different time points for recession depth in test group. The mean recession depth is more at baseline followed by 3 months and least at 6 months.

Table 6: Post hoc Bonferroni test representing mean difference different time points for recession depth in test group

| **Time points** | | **Mean Difference** | **Significance**  **(p)** |
| --- | --- | --- | --- |
| Baseline | 3months | 3.16 | **0.0001*** |
|  | 6months | 3.81 | **0.0001*** |
| 3months | 6months | 0.66 | **0.0001*** |

*Significance at p<0.05

A significant difference is present in test group for recession depth between:

Baseline and 3 months, baseline and 6 months, 3 months and 6 months.

Table 7: Mean difference between different time points for clinical attachment level in test group

| **Parameters** | **Time point** | **Mean** | **F value** | **Significance (p)** |
| --- | --- | --- | --- | --- |
| Clinical attachment level | Baseline | 4.71 | 2485.52 | **0.0001*** |
|  | 3months | 0.86 |  |  |
|  | 6months | 0.10 |  |  |

*Significance at p<0.05

A significant difference is present between different time points for clinical attachment level in test group. The mean clinical attachment level is more at baseline followed by 3 months and least at 6 months.

Table 8: Post hoc Bonferroni test representing mean difference different time points for clinical attachment level in test group

| **Time points** | | **Mean Difference** | **Significance**  **(p)** |
| --- | --- | --- | --- |
| Baseline | 3months | 3.85 | **0.0001*** |
|  | 6months | 4.61 | **0.0001*** |
| 3months | 6months | 0.76 | **0.0001*** |

*Significance at p<0.05

A significant difference is present in test group for clinical attachment level between:

Baseline and 3 months, baseline and 6 months, 3 months and 6 months.

Table 9: Mean difference between different time points for width of keratinized gingiva in test group

| **Parameters** | **Time point** | **Mean** | **F value** | **Significance (p)** |
| --- | --- | --- | --- | --- |
| Width of keratinized gingiva | Baseline | 3.51 | 209.62 | **0.0001*** |
|  | 3months | 4.18 |  |  |
|  | 6months | 5.00 |  |  |

*Significance at p<0.05

A significant difference is present between different time points for width of keratinized gingiva in test group. The mean width of keratinized gingiva is more at 6 months followed by 3 months and least at baseline.

Table 10: Post hoc Bonferroni test representing mean difference different time points for width of keratinized gingiva in test group

| **Time points** | | **Mean Difference** | **Significance**  **(p)** |
| --- | --- | --- | --- |
| Baseline | 3months | 0.67 | **0.0001*** |
|  | 6months | 1.49 | **0.0001*** |
| 3months | 6months | 0.81 | **0.0001*** |

*Significance at p<0.05

A significant difference is present in test group for width of keratinized gingiva between:

Baseline and 3 months, baseline and 6 months, 3 months and 6 months.

Table 11: Mean difference between different time points for recession width in test group

| **Parameters** | **Time point** | **Mean** | **F value** | **Significance (p)** |
| --- | --- | --- | --- | --- |
| Recession width | Baseline | 4.44 | 546.08 | **0.0001*** |
|  | 3months | 3.17 |  |  |
|  | 6months | 1.67 |  |  |

*Significance at p<0.05

A significant difference is present between different time points for recession width in test group. The mean recession width is more at baseline followed by 3 months and least at 6 months.

Table 12: Post hoc Bonferroni test representing mean difference different time points for recession width in test group

| **Time points** | | **Mean Difference** | **Significance**  **(p)** |
| --- | --- | --- | --- |
| Baseline | 3months | 1.23 | **0.0001*** |
|  | 6months | 2.78 | **0.0001*** |
| 3months | 6months | 1.50 | **0.0001*** |

*Significance at p<0.05

A significant difference is present in test group for recession width between:

Baseline and 3 months, baseline and 6 months, 3 months and 6 months.

Table 13: Mean difference between different time points for gingival thickness at 2mm, 4mm, and 6mm in test group

| **Parameters** | **Time point** | **Mean** | **Mean difference** | **t value** | **Significance (p)** |
| --- | --- | --- | --- | --- | --- |
| Gingival thickness at 2mm | Baseline | 1.29 | 0.96 | 31.139 | **0.0001*** |
|  | 6months | 2.25 |  |  |  |
| Gingival thickness at 4mm | Baseline | 1.20 | 1.03 | 40.515 | **0.0001*** |
|  | 6months | 2.23 |  |  |  |
| Gingival thickness at 6mm | Baseline | 1.32 | 0.95 | 8.435 | **0.0001*** |
|  | 6months | 2.27 |  |  |  |

*Significance at p<0.05

A significant difference is present between baseline and 6 months for gingival thickness in test group at 2mm, 4mm, and 6mm. The mean gingival thickness is more after 6 months compared to baseline at 2mm, 4m, and 6mm.

Table 14: Mean difference between different time points for periodontal probing depth in control group

| **Parameters** | **Time point** | **Mean** | **F value** | **Significance (p)** |
| --- | --- | --- | --- | --- |
| Periodontal probing depth | Baseline | 0.92 | 430.280 | **0.0001*** |
|  | 3months | 0.16 |  |  |
|  | 6months | 0.04 |  |  |

*Significance at p<0.05

A significant difference is present between different time points for periodontal probing depth in control group. The mean probing pocket depth is more at baseline followed by 3 months and least at 6 months.

Table 15: Post hoc Bonferroni test representing mean difference different time points for periodontal probing depth in control group

| **Time points** | | **Mean Difference** | **Significance**  **(p)** |
| --- | --- | --- | --- |
| Baseline | 3months | 0.76 | **0.0001*** |
|  | 6months | 0.88 | **0.0001*** |
| 3months | 6months | 0.12 | **0.0001*** |

*Significance at p<0.05

A significant difference is present in control group for probing pocket depth between:

Baseline and 3 months, baseline and 6 months, 3 months and 6 months.

Table 16: Mean difference between different time points for recession depth in control group

| **Parameters** | **Time point** | **Mean** | **F value** | **Significance (p)** |
| --- | --- | --- | --- | --- |
| Recession depth | Baseline | 3.59 | 2966.96 | **0.0001*** |
|  | 3months | 0.88 |  |  |
|  | 6months | 0.18 |  |  |

*Significance at p<0.05

A significant difference is present between different time points for recession depth in control group. The mean recession depth is more at baseline followed by 3 months and least at 6 months.

Table 17: Post hoc Bonferroni test representing mean difference different time points for recession depth in control group

| **Time points** | | **Mean Difference** | **Significance**  **(p)** |
| --- | --- | --- | --- |
| Baseline | 3months | 2.71 | **0.0001*** |
|  | 6months | 3.41 | **0.0001*** |
| 3months | 6months | 0.70 | **0.0001*** |

*Significance at p<0.05

A significant difference is present in control group for recession depth between:

Baseline and 3 months, baseline and 6 months, 3 months and 6 months.

Table 18: Mean difference between different time points for clinical attachment level in control group

| **Parameters** | **Time point** | **Mean** | **F value** | **Significance (p)** |
| --- | --- | --- | --- | --- |
| Clinical attachment level | Baseline | 4.50 | 3347.64 | **0.0001*** |
|  | 3months | 1.04 |  |  |
|  | 6months | 0.22 |  |  |

*Significance at p<0.05

A significant difference is present between different time points for clinical attachment level in control group. The mean clinical attachment level is more at baseline followed by 3 months and least at 6 months.

Table 19: Post hoc Bonferroni test representing mean difference different time points for clinical attachment level in control group

| **Time points** | | **Mean Difference** | **Significance**  **(p)** |
| --- | --- | --- | --- |
| Baseline | 3months | 3.46 | **0.0001*** |
|  | 6months | 4.29 | **0.0001*** |
| 3months | 6months | 0.83 | **0.0001*** |

*Significance at p<0.05

A significant difference is present in control group for clinical attachment level between:

Baseline and 3 months, baseline and 6 months, 3 months and 6 months.

Table 20: Mean difference between different time points for width of keratinized gingiva in control group

| **Parameters** | **Time point** | **Mean** | **F value** | **Significance (p)** |
| --- | --- | --- | --- | --- |
| Width of keratinized gingiva | Baseline | 3.42 | 109.51 | **0.0001*** |
|  | 3months | 3.97 |  |  |
|  | 6months | 4.53 |  |  |

*Significance at p<0.05

A significant difference is present between different time points for width of keratinized gingiva in control group. The mean width of keratinized gingiva is more at 6 months followed by 3 months and least at baseline.

Table 21: Post hoc Bonferroni test representing mean difference different time points for width of keratinized gingiva in control group

| **Time points** | | **Mean Difference** | **Significance**  **(p)** |
| --- | --- | --- | --- |
| Baseline | 3months | 0.55 | **0.0001*** |
|  | 6months | 1.11 | **0.0001*** |
| 3months | 6months | 0.56 | **0.0001*** |

*Significance at p<0.05

A significant difference is present in control group for width of keratinized gingiva between:

Baseline and 3 months, baseline and 6 months, 3 months and 6 months.

Table 22: Mean difference between different time points for recession width in control group

| **Parameters** | **Time point** | **Mean** | **F value** | **Significance (p)** |
| --- | --- | --- | --- | --- |
| Recession width | Baseline | 4.41 | 510.44 | **0.0001*** |
|  | 3months | 3.24 |  |  |
|  | 6months | 2.13 |  |  |

*Significance at p<0.05

A significant difference is present between different time points for recession width in control group. The mean recession width is more at baseline followed by 3 months and least at 6 months.

Table 23: Post hoc Bonferroni test representing mean difference different time points for recession width in control group

| **Time points** | | **Mean Difference** | **Significance**  **(p)** |
| --- | --- | --- | --- |
| Baseline | 3months | 1.17 | **0.0001*** |
|  | 6months | 2.28 | **0.0001*** |
| 3months | 6months | 1.12 | **0.0001*** |

*Significance at p<0.05

A significant difference is present in control group for recession width between:

Baseline and 3 months, baseline and 6 months, 3 months and 6 months.

Table 24: Mean difference between different time points for gingival thickness at 2mm, 4mm, and 6mm in control group

| **Parameters** | **Time point** | **Mean** | **Mean difference** | **t-value** | **Significance (p)** |
| --- | --- | --- | --- | --- | --- |
| Gingival thickness at 2mm | Baseline | 1.29 | 0.79 | 25.734 | **0.0001*** |
|  | 6months | 2.08 |  |  |  |
| Gingival thickness at 4mm | Baseline | 1.28 | 0.80 | 32.468 | **0.0001*** |
|  | 6months | 2.09 |  |  |  |
| Gingival thickness at 6mm | Baseline | 1.20 | 0.87 | 31.322 | **0.0001*** |
|  | 6months | 2.08 |  |  |  |

*Significance at p<0.05

A significant difference is present between baseline and 6 months for gingival thickness in control group at 2mm, 4mm, and 6mm. The mean gingival thickness is more after 6 months compared to baseline at 2mm, 4m, and 6mm.

Table 25: Mean difference between test and control group for different parameters

| **Parameters** | **Time point** | **Groups** | **N** | **Mean** | **Mean difference** | **t-value** | **Significance (p)** |
| --- | --- | --- | --- | --- | --- | --- | --- |
| Periodontal probing depth | Baseline | Test group | 108 | 0.81 | 0.11 | 2.572 | .011 |
|  |  | Control group | 108 | 0.92 |  |  |  |
|  | 3months | Test group | 108 | 0.13 | 0.04 | 1.105 | .270 |
|  |  | Control group | 108 | 0.16 |  |  |  |
|  | 6months | Test group | 108 | 0.02 | 0.02 | 1.516 | .131 |
|  |  | Control group | 108 | 0.04 |  |  |  |
| Recession depth | Baseline | Test group | 108 | 3.89 | 0.30 | 4.455 | .000 |
|  |  | Control group | 108 | 3.59 |  |  |  |
|  | 3months | Test group | 108 | 0.74 | 0.14 | 2.802 | **.006*** |
|  |  | Control group | 108 | 0.88 |  |  |  |
|  | 6months | Test group | 108 | 0.08 | 0.10 | 2.799 | **.006*** |
|  |  | Control group | 108 | 0.18 |  |  |  |
| Percentage root coverage | At 6 months | Test group | 108 | 97.10 | 1.74 | 1.69 | .091 |
|  |  | Control group | 108 | 95.37 |  |  |  |
| Clinical attachment level | Baseline | Test group | 108 | 4.71 | 0.21 | 2.557 | .011 |
|  |  | Control group | 108 | 4.50 |  |  |  |
|  | 3months | Test group | 108 | 0.86 | 0.18 | 2.749 | **.006*** |
|  |  | Control group | 108 | 1.04 |  |  |  |
|  | 6months | Test group | 108 | 0.10 | 0.12 | 3.184 | **.002*** |
|  |  | Control group | 108 | 0.22 |  |  |  |
| Width of keratinized gingiva | Baseline | Test group | 108 | 3.51 | 0.09 | .713 | .477 |
|  |  | Control group | 108 | 3.42 |  |  |  |
|  | 3months | Test group | 108 | 4.18 | 0.21 | 1.874 | .062 |
|  |  | Control group | 108 | 3.97 |  |  |  |
|  | 6months | Test group | 108 | 5.00 | 0.47 | 3.924 | **.0001*** |
|  |  | Control group | 108 | 4.53 |  |  |  |
| Recession width | Baseline | Test group | 108 | 4.44 | 0.04 | .401 | .689 |
|  |  | Control group | 108 | 4.41 |  |  |  |
|  | 3months | Test group | 108 | 3.17 | 0.07 | .857 | .392 |
|  |  | Control group | 108 | 3.24 |  |  |  |
|  | 6months | Test group | 108 | 1.67 | 0.45 | 4.974 | **.0001*** |
|  |  | Control group | 108 | 2.13 |  |  |  |

*Significance at p<0.05

A significant difference is present between the test group and control group for:

Recession depth at 3 months and 6 months (recession depth was more in control group than test group)

Clinical attachment level at 3 months and 6 months (clinical attachment level was more in control group than test group)

Width of keratinized gingiva at 6 months (width of keratinized gingiva was more in test group than in control group)

Recession width at 6 months (recession width was more in control group than in test group)

Table 26: Mean difference between test and control group for gingival thickness at 2mm, 4mm, and 6mm

| **Time point** | **Level** | **Groups** | **N** | **Mean** | **Mean difference** | **t-value** | **Significance (p)** |
| --- | --- | --- | --- | --- | --- | --- | --- |
| Baseline | At 2mm | Test group | 108 | 1.29 | 0.00 | 0.063 | 0.950 |
|  |  | Control group | 108 | 1.29 |  |  |  |
|  | At 4mm | Test group | 108 | 1.20 | 0.07 | 2.614 | **0.010*** |
|  |  | Control group | 108 | 1.28 |  |  |  |
|  | At 6mm | Test group | 108 | 1.32 | 0.12 | 1.072 | 0.285 |
|  |  | Control group | 108 | 1.20 |  |  |  |
| 6 months | At 2mm | Test group | 108 | 2.25 | 0.16 | 5.755 | **0.0001*** |
|  |  | Control group | 108 | 2.08 |  |  |  |
|  | At 4mm | Test group | 108 | 2.23 | 0.14 | 5.930 | **0.0001*** |
|  |  | Control group | 108 | 2.09 |  |  |  |
|  | At 6mm | Test group | 108 | 2.27 | 0.19 | 7.101 | **0.0001*** |
|  |  | Control group | 108 | 2.08 |  |  |  |

*Significance at p<0.05

A significant difference is present in gingival thickness between the test group and control group for:

At 4mm (gingival thickness is more in control group at 4mm compared to test group at baseline)

A significant difference is present in gingival thickness between the test group and control group for:

At 2mm, 4mm, and 6mm (gingival thickness is more in test group compared to control group at 6 months)

**GRAPHS**

Graph 1: Bar diagram representing mean periodontal probing depth at different time points in test group

Graph 2: Bar diagram representing mean recession depth at different time points in test group

Graph 3: Bar diagram representing mean clinical attachment level at different time points in test group

Graph 4: Bar diagram representing mean width of keratinized gingiva at different time points in test group

Graph 5: Bar diagram representing mean recession width at different time points in test group

Graph 6: Bar diagram representing mean gingival thickness at 2mm, 4mm, and 6mm for different time points in test group

Graph 7: Bar diagram representing mean periodontal probing depth at different time points in control group

Graph 8: Bar diagram representing mean recession depth at different time points in control group

Graph 9: Bar diagram representing mean clinical attachment level at different time points in control group

Graph 10: Bar diagram representing mean width of keratinized gingiva at different time points in control group

Graph 11: Bar diagram representing mean recession width at different time points in control group

Graph 12: Bar diagram representing mean gingival thickness at 2mm, 4mm, and 6mm for different time points in control group

Graph 13: Mean periodontal probing depth in test and control group at different time points

Graph 14: Mean recession depth in test and control group at different time points

Graph 15: Mean percentage root coverage in test and control group at different time points

Graph 16: Mean clinical attachment level in test and control group at different time points

Graph 17: Mean width of keratinized gingiva in test and control group at different time points

Graph 18: Mean recession width in test and control group at different time points

Graph 19: Mean gingival thickness in test and control group at different time points
